# O-36 - RE-INTRODUCTION OF BCG VACCINATION, OPPORTUNITIES AND CHALLENGES FOR PUBLIC HEALTH

**DOI:** 10.1093/pubmed/fdag046.032

**Published:** 2026-07-09

**Authors:** Mr Toney Thomas

**Affiliations:** Health Protection, HSE; RCSI, Faculty of Nursing and Midwifery

## Abstract

**Background:**

The universal BCG vaccination program ceased in Ireland (ROI) in 2015. However, with increasing incidence of Tuberculosis (TB), changing epidemiology and scientific evidence, policy makers prioritised re-introduction of BCG vaccine for select groups. Re-introduction of BCG vaccination in the ROI (2026) is presented.

**Objectives:**

To describe the phased re-introduction of BCG vaccination. To explore opportunities and challenges that deter or support BCG rollout. To catalogue methods employed to address gaps on safe BCG administration.

**Methods:**

BCG re-introduction planned in three phases, the eligible population in each phase defined. Efficacy of BCG vaccination to prevent TB, mostly among new-born to <5 years age group revisited. The need for Mantoux or IGRA test before BCG explored. The opportunities to prevent and early risk assessment of TB Infection and Disease among high-risk population considered. Interventions targeted to address gaps: knowledge and skills, workforce training, organisational preparedness on safe intra-dermal BCG vaccination.

**Results:**

BCG vaccination resources developed both for the public and health-care practitioners; developed competency-based intra-dermal (ID) injection training; gained accreditation of the training program; training scaled-up using 'Train the Trainer' method; supervised practice; declaration of ID injection competence; were methods and processes employed during the phase 1 BCG vaccination.

**Conclusions:**

The health-care reform in ROI and devolvement of operational service delivery to regions with structures evolving, posed numerous challenges on delivery of BCG vaccination programme. Central planning-empowerment-performance-achievement (PEPA) employed along with devolved BCG operational delivery to the regions facilitated a pragmatic solution. Ongoing program evaluation incorporated for varying needs especially using the learning to plan for later phases.

